# The Microscope in Medicine

**Published:** 1891-01-10

**Authors:** Frank J. Wethered


### THE MICROSCOPE IN MEDICINE.

By FRANK J. WETHERED, M.D.

### I.—INTRODUCTORY.

THE microscope in medicine has so much come into vogue of late years that it behoves every medical man to be thoroughly familiar with its uses. Could Hans Zansz, when he invented and made the first compound microscope at Middelburg in Holland, about 1590, have foreseen the perfection to which such instruments have now been brought, it would have indeed surpassed his wildest dreams. The cultivation of microscopical research has laid the basis of some of the most important branches of medical science, histology, physiological and pathological, and of later years the science which seems to bid fair to outshine all others in its far reaching and deep searching discoveries, *i.e.*, bacteriology. This latter study has always been regarded by some with great scepticism, but the famous discovery of to-day, by the world-known bacteriologist, Dr. Robert Koch, has completely, and once and for all, vindicated its claims, thus forming an epoch in medicine only equalled by Jenner's discovery of vaccination and Lister's introduction of antiseptics.

Information given us by the microscope is not only useful after the death of the patient, to which use it has generally been confined for the examination of dead tissues, but affords us the utmost practical help for the purpose of diagnosis during life. It is greatly to be regretted that pathology has not been recognised as pertaining to the study of disease in the living patient as well as after death. Paracelsus used to declare that there was an *anatomia vivorum*, and that this was a much more valuable science than the *anatomia mortuorum*, or dissection, of which he had a very low opinion; and though what Paracelsus understood by the "anatomy of the living" was pure fiction, the distinction, had it been founded on fact, would have been a very sensible one, and very applicable to the present subject.

What may be called "Clinical Pathology" is the bridge between clinical medicine and pathology, and for the study of it a thorough acquaintance with the microscope and with microscopical technique is absolutely necessary.

In this series of articles an attempt will be made to describe fully and minutely the work that has to be undertaken, not only in the pathological laboratory, but also, to some extent, by every practitioner. Great stress was laid on this point by Dr. Koch in his paper of November 14th in the *Deutsche Medicinische Wochenschrift*, wherein he states that in future great responsibility will rest upon every medical man, for the diagnosing of phthisis in its earliest stages, and that the main proof of this lies in the discovery in the sputum of the tubercle bacillus. This is an operation which can easily be performed in ten minutes, and is a process with which every doctor should make himself familiar.

In order to study this subject systematically, it is advisable to give some description first of all of the apparatus and reagents required.

For the purpose of examining pathological material, it is not at all necessary that a particular room be set apart, although if extended investigations are attempted, it is, of course, preferable. The chief thing requisite for comfort and exactness is that all the reagents and apparatus should be conveniently arranged in one place carefully protected from dust, and out of the way of accidental interference, so that specimens in course of preparation may be left without danger of disturbance.

If possible a room should be chosen with a north light, as it is important to secure a moderate, steady illumination, such as is reflected from a white cloud. For artificial light nothing equals an oil lamp—an ordinary paraffin lamp with a cardboard shade answers admirably. Various kinds of "microscopic lamps" are sold, which have in themselves no special advantages except for use with very high powers, when by means of a slit only a thin stream of light is allowed to fall upon the mirror, so producing more intense but localised illumination. For purposes of laboratory work at night, as well as for use with the microscope, the writer can confidently recommend "The Queen's" reading lamp, which burns the common colza, or any mineral oil, and is excellent as regards brilliancy and steadiness of light and economy in burning.

As a working bench an ordinary firm deal table with four legs is the best, which should be sufficiently large to hold all the bottles and apparatus, in addition to the space required for working. A very convenient size is 4 ft. by 2½ ft. Means should be at hand for easily securing a plentiful supply of water and a receptacle for waste material. A tap and sink are of course the best arrangement, but failing these a large jug and good-sized earthenware receiver answer all purposes admirably. A metal receiver will not do, as it is soon corroded by acids. For the same reasons if an ordinary sink is employed when using acids it should be frequently flushed by large quantities of water. All apparatus and reagents should be protected from dust either by being kept in a cupboard or carefully covered over when not in use. For the sake of handiness a cabinet may be employed so constructed as to fold into a convenient size when not required and open out something like an office desk, having the bottles, &c., at the back and a large space for working in front.

We now come to the important question, *i.e.*, the choice of a microscope.

Microscopes are distinguished as simple or compound. In the former, the rays which enter the eye of the observer come from an object brought near to it after refraction through either a single lens, or a combination of lenses, acting as a single lens, its action as a "magnifier" depending on its enabling the eye to form a distinct image of the object at a much shorter distance than would otherwise be possible. The latter consists of at least two lenses, so placed relatively to the object, to the eye, and to one another, that an enlarged image of the object, formed by the lens placed nearest to it (the "object-glass"), is looked at through the lens nearest the eye (the "eye-glass"), which acts as a simple microscope in magnifying it; so that the compound microscope may be described as a simple microscope used to look at an enlarged image of the object, instead of at the object itself.

Simple microscopes are only used for medical purposes in the form of "dissecting microscopes." As these, however are mainly used in histology, a description of them will not be entered into here.

It is extremely difficult to give advice to any one as to what sort of microscope he should buy, so much depending on the purse of the purchaser; and the work for which it is intended. For ordinary purposes a small "Hartnack" with powers Nos. 3 to 7 is sufficient, but if any bacteriological work is to be undertaken, even as simple as the examination for tubercle bacilli, a 12th oil immersion lens and an Abbé's condenser is requisite.

In purchasing a microscope it is advisable for an inexperienced person to seek advice from one who is well acquainted with the use of these instruments, as several points require close attention.

First, with regard to the stand. We would not advise a too large and cumbersome instrument, and by all means avoid a binocular. Many firms for the sale of scientific apparatus, make so-called "students' microscopes." Most of these are very good, and especially the more modern ones fitted for bacteriological work. They are all that can be desired or needed. Without wishing to particularise too much, the writer can confidently recommend those made by Pillischer, Swift, Baker, and Becker. The requisites for a good stand are weight and steadiness in the pedestal, which are best ensured by the horse-shoe or tripod pattern; the supporting column should be strong and rigid, while ample room should be allowed between the mirror and the sub-stage. The means for alteration in the angle of the tube are secured by a strong hinge placed just below the stage, and sufficient movement of the tube should be allowed so that the tube of the microscope can be drawn back into a perfectly horizontal position, this being required for use with the camera lucida.

The mirror should be reversible, having one side a plain surface and the other concave. The stage should be about three inches long by two and a-half wide, and perfectly fixed. Moveable stages are, in the opinion of the writer, not to be recommended, as the glass slide can be manipulated far more regularly and conveniently by means of the fingers.

